# The small GTPase RhoH is an atypical regulator of haematopoietic cells

**DOI:** 10.1186/1478-811X-6-6

**Published:** 2008-09-29

**Authors:** Florian Fueller, Katharina F Kubatzky

**Affiliations:** 1 Albert-Ludwigs-Universität, Institut für Experimentelle und Klinische Pharmakologie und Toxikologie, Albertstr. 25, 79104 Freiburg, Germany; 2 Ruprecht-Karls-Universität Heidelberg, Hygiene Institut, Abteilung für Hygiene und Medizinische Mikrobiologie, Im Neuenheimer Feld 324, 69120 Heidelberg, Germany; 3 Department of Pathology, University of Michigan, BSRB, 109 Zina Pitcher Pl., Ann Arbor, MI 48109-2200, USA

## Abstract

Rho GTPases are a distinct subfamily of the superfamily of Ras GTPases. The best-characterised members are RhoA, Rac and Cdc42 that regulate many diverse actions such as actin cytoskeleton reorganisation, adhesion, motility as well as cell proliferation, differentiation and gene transcription. Among the 20 members of that family, only Rac2 and RhoH show an expression restricted to the haematopoietic lineage.

RhoH was first discovered in 1995 as a fusion transcript with the transcriptional repressor LAZ3/BCL6. It was therefore initially named translation three four (TTF) but later on renamed RhoH due to its close relationship to the Ras/Rho family of GTPases. Since then, RhoH has been implicated in human cancer as the gene is subject to somatic hypermutation and by the detection of RHOH as a translocation partner for LAZ3/BCL6 or other genes in human lymphomas. Underexpression of RhoH is found in hairy cell leukaemia and acute myeloid leukaemia.

Some of the amino acids that are crucial for GTPase activity are mutated in RhoH so that the protein is a GTPase-deficient, so-called atypical Rho GTPase. Therefore other mechanisms of regulating RhoH activity have been described. These include regulation at the mRNA level and tyrosine phosphorylation of the protein's unique ITAM-like motif. The C-terminal CaaX box of RhoH is mainly a target for farnesyl-transferase but can also be modified by geranylgeranyl-transferase. Isoprenylation of RhoH and changes in subcellular localisation may be an additional factor to fine-tune signalling.

Little is currently known about its signalling, regulation or interaction partners. Recent studies have shown that RhoH negatively influences the proliferation and homing of murine haematopoietic progenitor cells, presumably by acting as an antagonist for Rac1. In leukocytes, RhoH is needed to keep the cells in a resting, non-adhesive state, but the exact mechanism has yet to be elucidated. RhoH has also been implicated as a regulatory molecule in the NFκB, PI3 kinase and Map kinase pathways. The recent generation of RhoH knockout mice showed a defect in thymocyte selection and TCR signalling of thymic and peripheral T-cells. However, RhoH-deficient mice did not develop lymphomas or showed obvious defects in haematopoiesis.

## Review

### RhoH is an atypical Rho GTPase

#### Small GTPases of the Rho family

The superfamily of mammalian Ras like proteins comprises over 150 members that can be subdivided into five main families, namely Ras, Rho, Rab, Arf and Ran and all control rather specifically one particular facet of cell metabolism. While Ras is an important regulator of proliferation [1], the main function of Rho proteins is the control of cell morphology [2,3]. Rho GTPases were first described in 1985 [4] and function as molecular switches that control and integrate a variety of signal transduction pathways by linking receptor-derived signals to downstream signalling proteins [2,5,6]. Rho proteins are present in lower eukaryotes such as slime mould [7] and yeast [8] and in mammals [9]. Twenty mammalian members of the Rho GTPases have been identified so far [10], which can be subdivided into 8 distinct subgroups [11]. They share a high degree of sequence similarity and the individual members are around 50% homologous to each other [12]. Only two members of the Rho family, Rac2 and RhoH, are specifically expressed in haematopoietic cells [13,14].

As is the case for most of the other Ras-like GTPases, Rho proteins are monomeric 20–30 kDa GTP binding proteins that act as molecular switches. They are turned on when bound to GTP and switched off when bound to GDP [12]. Three main classes of interacting proteins actively control this cycling. Guanine nucleotide exchange factors (GEFs) or Dbl homology proteins catalyse the exchange of GDP by GTP and therefore act as positive regulators [15]. GTPase activating proteins (GAPs) on the other hand enhance the intrinsic GTPase activity leading to the hydrolysis of GTP to GDP [16]. Only three proteins are known of the third class of regulators. These are the GDP dissociation inhibitors (GDI) that keep the GTPase in an inactive state in the cytosol [2,3]. In the GTP-bound state, the GTPase is in its active conformation and interacts with a variety of downstream effectors, such as protein kinases, lipid kinases, phospholipases C and D as well as several adaptor proteins [17,18]. However, like Rnd1, RhoE/Rnd3 and probably RhoBTB proteins, RhoH is GTPase-deficient [13] and therefore belongs to the subset of atypical GTPases. Its activity is modulated by interaction with other Rho GTPases [19,20], transcriptional regulation [19] or phosphorylation [21] rather than through the classical cycling process and modulation by effector proteins.

**Figure 1:**
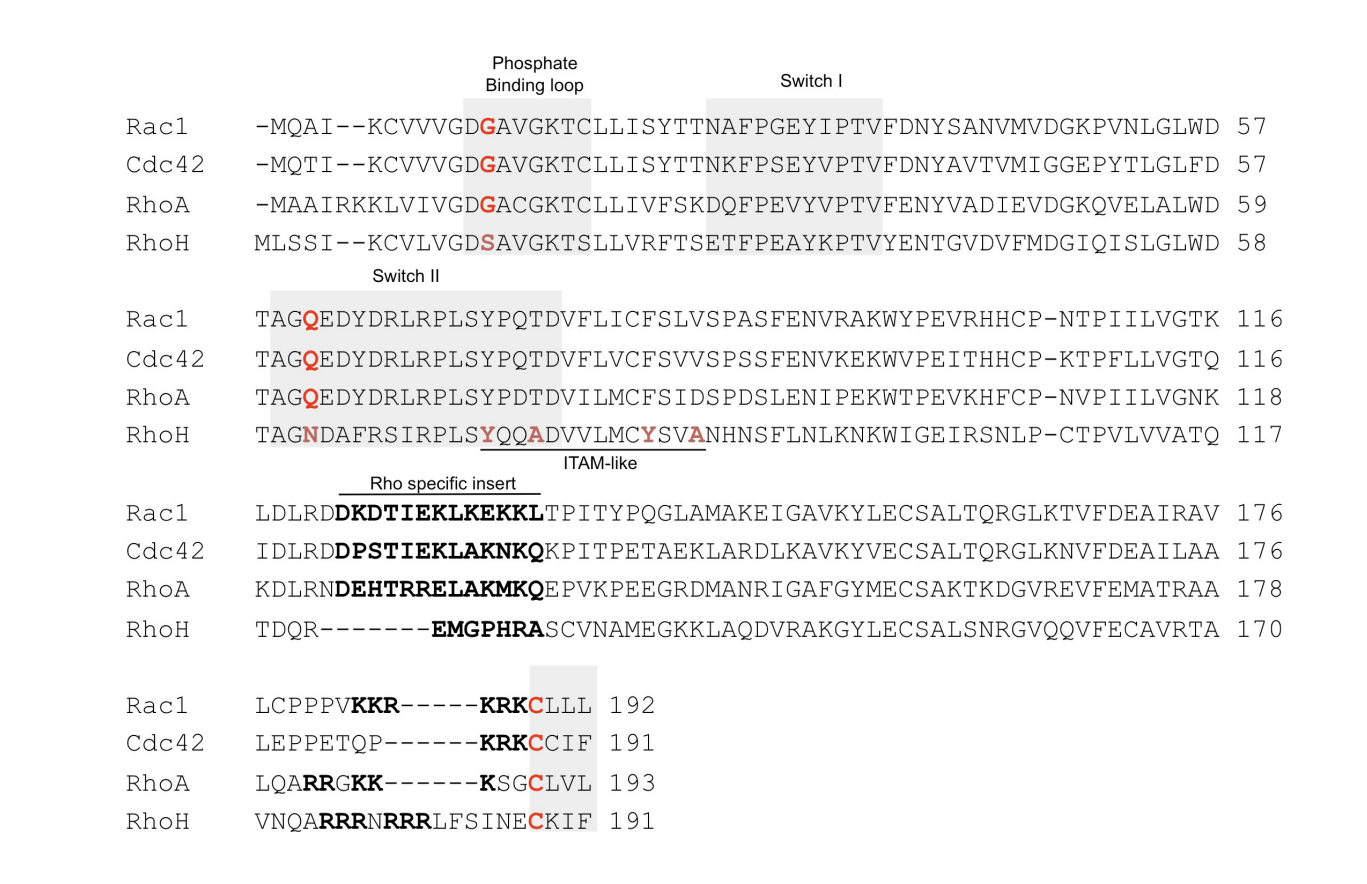
**Alignment of the most prominent human Rho GTPases**. RhoA, Rac1, Cdc42 and RhoH were aligned using the ClustalW alignment program [71]. Conserved residues are presented in red and RhoH specific amino acids are depicted in blue. The ITAM-like motif in RhoH comprising the sequence YxxA(X)_6_YxxA is underlined. Gray-shaded boxes mark the phosphate-binding loop, and switch I and switch II regions, respectively. The Rho family specific insertion motif that is absent in the other members of the Ras superfamily, is shown in bold typing.

Structurally, all Rho GTPases contain a Rho family specific, exposed insert of 13 amino acids that has a function in binding to effectors and regulators [22] (see Figure 1). In RhoH however, the insert consists of only 7 amino acids, which is another atypical feature of that protein. The highest sequence similarity within Rho GTPases is found for the central GTP binding motifs while the C-terminal sequences are more divergent [12]. Figure 1 gives an overview of the structural elements important for signalling for RhoA, Rac1, Cdc42 as well as RhoH. While the switch I and switch II regions constitute the conformational difference between the active, GTP-bound and the inactive, GDP-bound state (grey shaded boxes), the phosphate binding loop is essential for the GTPase activity of the protein. Like in Ras, replacement of the conserved glycine G12 in the phosphate-binding loop (marked in red within the grey shaded box) renders Rho GTPases enzymatically inactive. In RhoH and RhoE, another constitutively active Rho family member, this glycine is replaced by a serine. Another key residue for intrinsic and GAP mediated GTPase activity is glutamine Q61 (in Ras and Cdc42 or Q63 in RhoA) located within the switch II region that is replaced by an asparagine in RhoH (shown in red).

**Figure 2:**
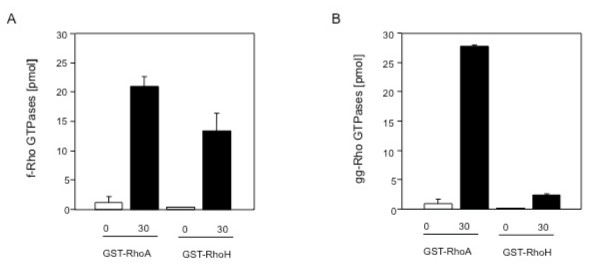
**Rho GTPases can be isoprenylated at their C-terminal CaaX box**. The CKIF motif in human and mouse RhoH is a target for the *in vitro *modification by A) farnesyl-transferase and B) geranylgeranyl-transferase. The isoprenylation reaction was performed as described elsewhere [23].

The C-terminus of Rho GTPases has a function in mediating interactions with the membrane through its polybasic motif and more importantly, by the CaaX motif. Figure 1 shows the basic residues in bold printing and the conserved cysteine in red. This cysteine can be modified post-translationally by prenylation through transfer of a geranylgeranyl or farnesyl lipid moiety to interact with the plasma membrane. After the prenylation reaction, the aaX tripeptide is clipped off and the cysteine's free carboxylate anion gets methylated [23]. RhoH contains a CKIF motif and here we present biochemical data showing that RhoH is modified *in vitro *by farnesyl-transferase and to a small extent by geranylgeranyl-transferase (Figures 2A and 2B, respectively). During the preparation of this manuscript, the Der group showed that dual treatment of RhoH expressing cells with farnesyl-transferase and geranylgeranyl-transferase inhibitors caused relocalisation of RhoH to the cytoplasm [24]. However, they did not include biochemical data in their study as we present them here.

Phylogenetically, most Rho clusters seem to have already emerged in chordates, however, RhoH is only found in vertebrates [25]. Boureux et al., who investigated the evolution of Rho proteins, speculate that the RHOH gene might have been obtained by horizontal gene transfer, for example through retroviral integration. This hypothesis is supported by the fact that in contrast to other Rho family members, the coding region of RHOH is not split by introns. In addition, RhoH is found in all species as a single member whereas other Rho subgroups comprise at least two members in vertebrate genomes.

In addition to their well-described role as regulators of cell morphology, a number of Rho proteins also have important functions in the process of haematopoiesis (reviewed in [26]), although only Rac2 and RhoH are specifically expressed in haematopoietic tissue.

RhoH is thought to mainly act as a negative regulator for such diverse processes as proliferation, survival, migration and engraftment of haematopoietic progenitor cells [27] and was proposed to be a negative regulator for growth and actin based functions through suppression of Rac signalling. Very recently, the generation of RhoH-deficient mice allowed to study the function of RhoH in more detail. RhoH null mice are viable with no obvious defects in haematopoiesis and RhoH seems to be dispensable for the development of myeloid, erythroid and B-cells [28]. On the other hand, animals showed impaired T-cell differentiation due to defective T-cell receptor signalling [21,28], as RhoH is necessary for the production and survival or migration of T-cells.

#### RhoH is a Rho protein with atypical properties

Thirteen years ago, Dallery and co-workers set out to identify new potential oncogenes by cloning chromosomal translocation breakpoints that were found in human lymphomas [13]. They had previously shown that the zinc finger protein and translational repressor LAZ3/BCL6 is disrupted in non-Hodgkin lymphomas. Although these 3q27 chromosomal abnormalities mainly lead to rearrangements with immunoglobulin genes, other LAZ3/BCL6 rearrangements were also found to occur [29]. One recurrent translocation in the non-Hodgkin lymphoma cell line VAL was a t(3;4)(q27;p11) rearrangement that disrupted LAZ3/BCL6 in the first intron (see next paragraph). Subsequent sequencing of the LAZ3/BCL6 containing mRNA allowed them to discover a chimeric transcript that contained a previously unknown small G like protein, which they termed "translation three four" (TTF) [13]. Later on, more accurate FISH analysis corroborated RHOH as a LAZ3/BCL6 translocation partner and placed RHOH on the short arm of chromosome 4, band p13 [30].

The initial characterisation showed that RhoH was a 191 amino acid containing protein and a novel homologue of the Ras family. While it had 27% identity to H-RAS it showed up to 45% identity with members of the Rho family. It also contained the Rho typical insert motif, although shortened to 7 amino acids, which confirmed that classification. However, the absence of residues crucial for the catalytic activity of the proteins (see Figure 1) indicated that RhoH might have unusual properties. In addition, it was found that the 2.2 kb RhoH transcript is expressed only in haematopoietic cells while no expression in other organ tissues was detectable [13].

Investigation of the structural characteristics of the 35 kb spanning RHOH revealed an unusual organisation of its exons [31]. First of all, RHOH contains one intronless coding region, two major (1a and 1b) and three minor (X1, X2, X3) non-coding exons. The open reading frame comprises the complete coding sequence. This is uncommon as the coding region of most other Rho GTPases is interrupted by introns [10]. Interestingly, all currently described translocations or mutations of RHOH affect its 5' regulatory region but not the coding exon. Second, the authors discovered a great heterogeneity in the 5'-untranslated regions of mRNAs from different haematopoietic lineages, especially between B and T-cells. Alternative splicing of some 5' exons caused different transcription initiation sites [31]. This complex organisation of the RHOH 5' region is unusual for a Ras related gene and is reminiscent of cytokine or cytokine receptor genes. It is perceivable that this atypical feature plays a role in regulation of RhoH signalling through differential expression.

### RHOH is a target for genetic alterations

#### Introduction

**Figure 3:**
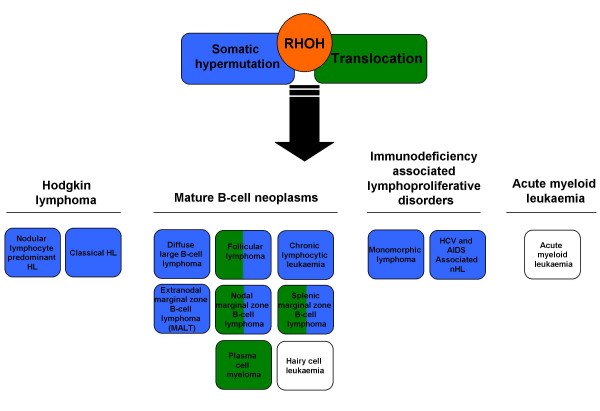
**RhoH in Cancer**. The two major mechanisms that link RhoH to cancer are aberrant somatic hypermutation and the formation of fusion transcripts with genes such as the transcriptional repressor LAZ3/BCL6. Both mechanisms have been found in a variety of human cancers and the figure summarises the current knowledge by sorting the described cancers corresponding to their WHO classification [36]. Cancers involving somatic mutation of the RHOH gene are highlighted in blue, while cancers, where a fusion of RhoH was detected, are presented in green. Cases of leukaemia characterised by underexpression of RhoH caused by an unknown mechanism are shown in white.

Since the original description of RhoH in a t(3;4)(q27;p13) translocation with LAZ3/BCL6 in a non-Hodgkin lymphoma cell line [13], alterations to the RHOH gene have been found in a number of different human cancers. The current literature recognises two main mechanisms that describe the cause for these alterations. Although there are reports where structural changes in the 4p13 region could be linked to translocation of the RHOH gene [32-34], most of the cases described so far show that RHOH is a hypermutable gene locus and targeted by aberrant somatic hypermutation [35]. Figure 3 categorises the described events according to the WHO classification of lymphoid tumours [36]. The four types of haematopoietic tumours involved are immunodeficiency associated lymphoproliferative disorders, Hodgkin lymphomas, mature B-cell neoplasms and acute myeloid leukaemia. Diseases involving RHOH translocations are shown in blue, while the green colour indicates aberrant somatic hypermutation of the RHOH gene. Leukaemia associated with an underexpression of RHOH mRNA are depicted without colour, as the mechanism of deregulation has not yet been established.

#### Lymphomas involving RHOH gene rearrangements

Reciprocal chromosomal translocations with oncogenes are found in a number of non-Hodgkin lymphomas. The occurring translocations are rather specific for the type of lymphoma and they are often the primary event causing the disease. Follicular lymphoma (FL) for example, is associated with a translocation involving the BCL2 gene. However, rearrangements involving LAZ3/BCL6, eventually interfering with LAZ3/BCL6 protein expression, are also found [37]. Bcl6 is a zinc finger protein known to act as a sequence specific transcriptional repressor; it plays an important role in the maturation of B-cells and high Bcl6 expression is considered to be of favourable prognostic value [38,39]. While a BCL2 translocation is found in 85% of FL cases and can be considered the primary cause of the disease, the t(3;4)(q27;p13) translocation between LAZ3/BCL6 and RHOH is often a secondary event [40]. The breakpoint and gene orientation indicates that the gene fusion places LAZ3/BCL6 under the control of the RHOH promoter sequence instead of the expression of a fusion protein. Reciprocally, RHOH is placed under control of the LAZ3/BCL6 promoter. This results in the transcriptional deregulation of both genes [32].

The importance of LAZ3/BCL6 translocations was investigated by Akasaka et al. [41]. The authors found that in the course of transformation from the rather indolent follicular lymphoma into the more aggressive diffuse large B-cell lymphoma, LAZ3/BCL6 translocations with RHOH were widespread. It is therefore possible that RHOH plays a role in the pathogenesis of follicular lymphoma and it may serve as a possible marker for those lymphomas that have a high potential to transform.

A different RHOH translocation was found in a case of multiple myeloma (or plasma cell myeloma). Chromosomal abnormalities and translocations seem to increase in pathogenesis in this bone marrow based plasma cell neoplasm [32]. In this patient only one abnormality was found involving a t(4;14)(p13;q32) translocation, resulting in a rearrangement of the RHOH gene with the IGH gene, although the corresponding fusion transcript could not be detected. Two other cases of a t(4;14)(p13;q32) translocation have been reported to date; one in a case of nodal marginal lymphoma [33] while the other involves a case of splenic lymphoma [34]. These data suggest that rearrangements involving RHOH are a recurrent abnormality in B-cell malignancies. However, it has not been investigated whether RHOH rearrangements have prognostic implications.

#### RHOH is a target for aberrant somatic hypermutation

Recently, a novel class of genetic aberration called aberrant somatic hypermutation (ASHM) has been described [35]. Somatic mutation is a physiologic process that enables B-cells to enhance antibody affinity for a specific antigen through nucleotide substitutions within the immunoglobulin variable genes of germinal centre B-cells [42]. However, this hypermutation activity may also aberrantly target other genes, among them a number of well-known proto-oncogenes such as PIM1, MYC, PAX5 and also RHOH. Interestingly, these genes are also susceptible to the formation of translocations which suggests that the process of hypermutation could also facilitate the formation of double-strand breaks [43]. Aberrant somatic mutation of RHOH has originally been described for diffuse large B-cell lymphoma (DLBCL), a group of aggressive lymphomas with heterogeneous clinical outcome [36]. In DLBCL, around 50% of the cases were found to harbour aberrant mutations for PIM1, PAX5, MYC and RHOH, respectively [35]. Since this initial observation, several similar studies on various lymphoid tumours were performed. Table 1 summarises the current knowledge.

**Table 1:** Aberrant somatic hypermutation of RHOH in human cancers.

**Cancer**	**Type and number of samples**	**ASHM Frequency**	**Remarks**	**Mutation Frequency for RhoH**	**Ref**
Hodgkin lymphoma (HL)	Nodular lymphocyte predominant HL (n = 10) Classic HL (n = 9)	80% 55.5%		11.1% 0%	[51]
Mature B-cell neoplasms	Diffuse large B-cell lymphoma (n = 39)	>50%		46%	[35]
Mature B-cell neoplasms	Nodal marginal zone lymphoma and Splenic marginal zone lymphoma (n = 55)	13%	Not c-MYC	3.6%	[44]
Mature B-cell neoplasms	Mucosa-associated lymphoid tissue lymphoma (n = 17) Extranodal diffuse large B-cell lymphoma (n = 17)	76.5% 100%		11.8% 47.1%	[45]
Mature B-cell neoplasms	Primary CNS lymphoma (n = 10)	90%		70%	[46]
Mature B-cell neoplasms	Primary cutaneous large B-cell lymphoma, leg type (n = 13) Primary cutaneous follicle centre lymphoma (n = 19)	54% 53%	Not PIM1	30.7% 10.5%	[47]
Mature B-cell neoplasms	Follicular lymphoma Diffuse large B-cell lymphoma (n = 9) Chronic lymphocytic leukaemia Diffuse large B-cell lymphoma (n = 9)	33.3% 55.5% 11.1% 22.2%		33% 11.1%	[48]
Mature B-cell neoplasms	Chronic lymphocytic leukaemia (n = 15) Diffuse large B-cell lymphoma (n = 13) Prolymphocytic transformation (n = 8)	26.7% 100% 100%	Not PIM1	0% 46.1% 25%	[49]
Mature B-cell neoplasms	Diffuse large B-cell lymphoma (n = 100)	>50%		35%	[50]
Mature B-cell neoplasms	Mediastinal B-cell lymphoma (n = 6)	100%	Not PIM1	66%	[72]
Mature B-cell neoplasms	Low grade follicular lymphoma (n = 32) Transformed follicular lymphoma (n = 26)	75% 77%		16% 31%	[73]
Immunodeficiency-associated lympho-proliferative disorders	Post-transplant LD (n = 21) Polymorphic PTLD (n = 8) Monomorphic PTLD (n = 12)	45% 12.5% 66.7%		20% 20% 40%	[52]
Immunodeficiency-associated lympho-proliferative disorders	AIDS-associated non-Hodgkin lymphoma (n = 39)	48.7%		23.1%	[53]
Immunodeficiency-associated lympho-proliferative disorders	HCV-associated non-Hodgkin lymphoma (n = 9)	31%		13%	[54]

Summary of currently available data on RHOH as a target for ASHM. The general ASHM frequency is defined by the frequency for mutations in at least one of the following genes: PIM1, PAX5, MYC and RHOH, except where otherwise indicated.

In mature B-cell neoplasms, the occurrence of ASHM and the mutation frequency of RHOH vary. While some cancers have low rates of ASHM in general [44], others with frequent ASHM do not include RHOH as a target [45]. However, indolent forms of a certain cancer often have lower mutation frequencies for RHOH than more aggressive forms. One example is primary CNS lymphoma, a subtype of DLBCL that has much poorer prognosis compared to DLBCL (70% mutation frequency in RHOH vs. 46%) [46]. Similar results were obtained for patients with two subtypes of primary cutaneous lymphoma, the indolent primary cutaneous follicle centre lymphoma (PCFCL) and the more aggressive primary cutaneous large B-cell lymphoma, leg type (PCLBCL) [47]. ASHM can also occur during progression of the disease as described by Rossi and Reiniger and their co-workers [48,49]. Here, the accumulation of novel mutations in RHOH and other gene loci (PIM1, PAX5, MYC) was found to be associated with transformation of follicular lymphoma and chronic lymphocytic leukaemia to DLBCL. To evaluate the potential role of RhoH as a prognostic marker, samples from 100 patients with previously untreated DLBCL were examined [50]. However, neither the overall survival nor the disease-free survival rate could be linked to hypermutation of the RHOH gene.

Although Hodgkin lymphomas (HL) are targeted frequently by ASHM (55–80%), the RHOH gene is not mutated at high frequency [51]. Both subtypes of Hodgkin lymphomas are rarely fatal due to effective treatment, supporting again the notion that ASHM of RHOH mainly occurs in aggressive types of lymphomas.

The third group of lymphoid tumours, where abnormalities of RHOH are reported, are immunodeficiency-associated disorders such as the B-cell post-transplant lymphoproliferative disorder (PTLD). PTLD is a lymphoid proliferation or lymphoma that may result from immunosuppression after receiving solid organ or bone marrow allografts. While the polymorphic form of the disorder rarely undergoes ASHM (12.5%), the monomorphic form, representing a progression of the disease, had ASHM in the majority of cases investigated (66.7%) [52]. This is similar to the aforementioned results obtained for the progression from the more indolent follicular lymphoma and chronic lymphocytic leukaemia to diffuse large B-cell lymphoma [48,49]. The mutation frequency for RHOH in monomorphic PTLD was found to be 40% and therefore nearly as high as in DLBCL. In AIDS-associated non-Hodgkin lymphoma, another immunodeficiency-associated lymphoma type, aberrant SHM may contribute to the pathogenesis of that disease [53]. In HCV-positive and HIV-positive patients alterations of RHOH, however, were lower than in patients negative for HCV or HIV [53,54].

#### Reduced expression of RHOH in cancer

Two recent publications highlight the fact that RhoH is underexpressed in hairy cell leukaemia (HCL) and acute myeloid leukaemia (AML), respectively [55,56].

HCL is a rare disease, comprising only 2% of lymphoid leukaemia cases. The origin of the disease is not known and effective treatment is not available [57]. The disease is diagnosed by the overexpression of the myeloid specific marker CD11c. Lymphocytes isolated from HCL patients demonstrate app. 3–4 fold lower RHOH mRNA expression compared to a variety of other diseases of the lymphoid system, such as Burkitt lymphoma, plasmocytoma or T-cell acute lymphoblastic leukaemia. The lower mRNA expression was found to be caused by transcriptional repression of the RHOH promoter [55]. HCL cells show a Ras-induced, increased activity of the transcription factor AP-1 that results in the upregulation of the AP-1 dependent gene CD11c [58]. *In vitro *reconstitution of RhoH expression was sufficient to repress CD11c surface expression.

CD11c is the integrin alpha X subunit that together with the beta2 chain acts as a leukocyte specific integrin. So the authors asked whether RhoH plays a role in intercellular adhesion. Indeed, cells reconstituted with RhoH showed decreased cell proliferation and homotypic and heterotypic adhesion, respectively. In a transwell assay, the authors could demonstrate that RhoH reconstitution reduced transendothelial migration, a central process in the pathogenesis of the disease. In an *in vivo *xenograft model, RhoH reconstitution leads to the inhibition of malignant progression. The tumour burden of the mice was reduced by 70% suggesting that elevated levels of RhoH protected the animals against mortality [55].

The expression level of RhoH was also investigated in bone marrow samples from AML patients and underexpression of RHOH on the mRNA level was found to be a prognostic marker for bad prognosis, overall survival as well as disease-free survival [56]. This is in contrast to results published by the same group on the impact of aberrant SHM of RHOH in diffuse large B-cell lymphoma, where such a link was not detectable [50]. The authors also provide a first insight into the molecular mechanism that might play a role in resistance to chemotherapy in AML patients. Rac1-induced activation of its downstream effector p21-activated kinase 1 (PAK1) leads to phosphorylation of the anti-apoptotic protein Bad on serine S75. This protects human lymphoma cells from drug-induced caspase activation and subsequent apoptosis [59]. While overexpression of RhoH suppresses Rac1 activity thus leading to increased apoptosis (see following paragraph) [27], low expression levels of RhoH might increase Rac1-induced protection from apoptosis, drug resistance and cause bad prognosis.

It is to be expected that RhoH expression levels can modulate the activity of other proteins involved in cell survival and proliferation as well. It will therefore be of crucial importance to gain a better understanding of RhoH as a modulator of signalling cascades in order to better understand its biological implications in cancer patients.

### RhoH in signal transduction

#### Aspects of RhoH signalling

In comparison to the number of studies on the putative role of RhoH in cancer, only a very limited number of publications deal with the function of RhoH as a signalling molecule. This can be attributed in part to the fact that all mutations of the RHOH gene were found to occur in the non-coding part of the gene. This suggests that in human haematological malignancies the absence of RhoH is problematic rather than activating mutations or overexpression of the protein. The recent finding that overexpression of RhoH in hairy cell leukaemia reduces the transformation potential [55] might stimulate a new interest in the functioning of RhoH on a molecular level.

Seven years after the initial discovery of RhoH, the first study that described several aspects of the signalling of RhoH in various haematopoietic cell types was published [19]. Due to the mutation of the conserved residues G13 and Q61 which are essential for hydrolysis of GTP, it was proposed that RhoH has no intrinsic GTPase activity [12,13]. In a nucleotide dissociation assay it was shown that although RhoH is able to bind GTP rapidly, it does not hydrolyse the bound GTP to GDP [19]. It is possible that RhoH might not have intrinsic GTPase activity but can substitute for that defect by binding to a GAP that provides the critical residues necessary for catalytic activity. This was described for Rap that hydrolyses GTP when RapGAP1 provides the catalytic arginine [60]. The authors investigated whether the RhoGAP p50 which acts as a GAP for RhoA, Rac and Cdc42 might enable RhoH to hydrolyse GTP, but found it not the case. Nevertheless, it is still possible that a RhoH specific GAP exists and hasn't been identified yet. The authors also tested another class of Rho regulatory molecules, the GDIs. These molecules inhibit dissociation of GDP and keep Rho GTPases in their inactive form in the cytoplasm. Their finding that the three Rho GDIs 1, 2 and 3 can all interact with RhoH is a matter of controversy, as others report that this interaction was only marginally detectable [61].

As Rho GTPases characteristically play a function in cell morphology and the organisation of the actin cytoskeleton, the effects of RhoH expression on actin polymerisation were studied in non-haematopoietic NIH 3T3 and MDCK cells. The authors describe RhoH to be diffusely distributed in the cytoplasm and they saw no difference in the morphology of PDGF stimulated cells in the presence or absence of RhoH overexpression [19]. However, more recent studies using bone marrow from RhoH-deficient mice or haematopoietic cells transduced with RhoH showed that RhoH does have an effect in regulation of the cytoskeleton in haematopoietic cells, if only as an antagonist of another Rho GTPase, Rac1 [20,27]. SDF-1α is an important chemoattractant that induces migration of haematopoietic progenitor cells [62]. It induces a polarised rim in haematopoietic cells through activation of Rac1. This enables the cells to generate membrane protrusions needed for cell migration. RhoH-transduced cells, however, showed reduced Rac1 activity, reduced polarised cortical F-actin staining and less cell migration in response to SDF-1α [27]. UsingPηoH-δεϕιχιεντmice, it was investigated in more detail how RhoH modulates migration and Rac1-promoted cortical rim polarisation [20]. The authors were able to show that the C-terminal prenylation motif of the CaaX box is an important regulator of the inhibitory effect of RhoH on Rac. In myeloid 32D cells transduced with RhoH, RhoH was localised at the membrane while deletion of the CaaX box led to an exclusively cytoplasmic localisation. Since active GTP-bound Rac is localised at the membrane through interaction of its CaaX box with lipid rafts, only the membrane-localised form of RhoH is able to downregulate Rac1 efficiently [20].

The antagonistic function of RhoH had already been described in the initial study of Li et al., where RhoH was shown to reduce the RhoA, Rac1 or Cdc42-induced activation of the transcription factor NFκB. Jurkat cells stimulated with TNFα show decreased NFκB activity in the presence of RhoH [19]. Initial experiments showed that IKKβ expression that is necessary for NFκB activation is suppressed in the presence of high RhoH amounts, presumably because RhoH inhibits IκB degradation. In addition, it was investigated whether RhoH could activate the MAP kinases p38, JNK or Erk. Again, as with NFκB, RhoH overexpression did not induce activation of any of these proteins itself, but rather acted as a specific negative regulator for Rac and RhoA-induced p38 activity. It was hypothesised that the inhibition does not affect Rac1 or the Rac1 GEF TIAM-1, but presumably occurs on the level of downstream effectors. However, the significance of these experiments performed in HEK293 cells has not been corroborated by other studies in a more physiologic experimental setup.

Since RhoH is GTPase-deficient, other mechanisms must be available to regulate the activity or function of the protein, one being the possibility of transcriptional regulation. Li et al. tested a couple of stimuli on Jurkat cells and reported that PMA reduces endogenous RhoH transcripts after 60–80 min by 80% of the baseline level, while TNFα treatment did not act as a regulator of RhoH mRNA levels [19]. So far no comprehensive study has been performed on potential inducers or regulators of RhoH expression though it can be anticipated that much can be learned from the characterisation of these molecules.

In a study aimed at identifying regulators of lymphocyte adhesion factor 1 (LFA-1), RhoH was found to be of importance [63]. LFA-1 is a β2 integrin expressed on leukocytes. In lymphocytes, LFA-1 is important for diverse processes such as migration, antigen presentation or cytotoxicity. On resting leukocytes, LFA-1 is in its non-adhesive state but appropriate stimulation renders LFA-1 active thereby increasing the adhesiveness of cells. By searching for a deregulated LFA-1 phenotype using a retroviral insertion mutagenesis approach, Cherry et al. found that a decrease of RhoH expression activated LFA-1, thus rendering the leukocytes adhesive and therefore active. However, when the authors tried to decrease LFA-1 mediated adhesion by overexpressing RhoH, no change in adhesion was detectable. This led the authors to the hypothesis that endogenous RhoH is already maximally active, i.e. inhibitory for LFA-1, in resting cells. The authors hypothesise that RhoH provides an inhibitory signal that downregulates activation pathways in resting leukocytes. Recently, the Brakebusch group investigated whether regulation of LFA-1 mediated adherence by RhoH can also be observed in T-lymphocytes, but found that not to be the case [28].

Supporting the theory that RhoH acts as a negative regulator of Rac signalling and other signalling cascades, it was found that RhoH negatively influences proliferation, migration and engraftment of haematopoietic bone marrow derived progenitor cells [27]. To assess the influence of RhoH on haematopoietic progenitor cells, cells were retrovirally transduced with wildtype RhoH and tested *in vitro *for their ability to respond to a cytokine stimulus [27]. In response to stem cell factor (SCF), high RhoH expression levels reduced growth and formation of myeloid colonies compared to control cells by half, due to an increase in apoptotic cells. In order to investigate the role of RhoH in engraftment of haematopoietic cells, RhoH transduced cells were injected into lethally irradiated mice and haematopoietic reconstitution was examined six month post transplantation. Although RhoH transduced donor cells allowed normal distribution of all lineages, the percentage of engraftment was severely reduced for RhoH overexpressing cells (app. 50%). These data indicated that RhoH overexpression might impair long-term reconstitution of haematopoietic progenitor cells. Conversely, a knockdown of RhoH stimulated proliferation and survival, as well as SDF-1α mediated migration *in vivo *[27]. However, the phenotype of the RhoH-deficient mice does not suggest that the mice have a defect in maintenance of haematopoietic progenitors [21,28]. Nevertheless, the recent finding that RHOH is underexpressed in certain types of leukaemia implies that RhoH expression levels are a crucial factor in protection from the development of haematopoietic malignancies [56,57].

#### RhoH as a regulator of T-cell signalling

Because RhoH is expressed strongest in T-cells, it was tested early on whether differentiation of T-cells into Th1 and Th2 subsets leads to varying RhoH expression patterns [19]. Indeed, the level of RhoH is app. 3 times higher in the Th1 subset. Re-stimulation of cells on day 3 using anti-CD3 led to a decrease of RhoH expression in Th1 cells whereas no obvious changes seemed to have occurred in the Th2 cells.

Using RhoH-deficient mice, the analysis of the function of RhoH in T-cells was investigated in more detail. Two groups independently showed that RhoH acts as a positive regulatory factor for thymocyte selection and T-cell receptor signalling [21,28]. RhoH-deficient mice did not develop lymphomas and had no obvious defects in HSC maintenance, but they showed impaired T-cell differentiation attributed to defective T-cell receptor signalling. T-lymphocyte maturation is a tightly regulated process involving gene rearrangement of TCR genes, negative and positive selection and eventually proliferative expansion of selected cells. During this maturation, several checkpoints can be distinguished: β-selection through the pre-TCR complex and negative and positive selection, controlled by the fully assembled TCRαβ antigen complex, respectively [64].

RhoH-deficient mice were found to have abnormally low levels of T-lymphocytes and the cellularity of their thymus was app. 60% lower than in control animals, due to reduced proliferative cell expansion. In mutant mice, the number of double negative thymocytes was reduced by 60%, indicating that the development between double negative (DN) to double positive (DP) stages was disturbed; this was also reflected in an elevated number of CD44^- ^CD25^+ ^DN3 cells. During thymocyte positive selection, TCR mediated signalling results in upregulation of markers for positive selection, such as CD5 and CD69. In RhoH-deficient mice the expression of these markers was reduced, suggesting that RhoH plays a role in thymocyte positive selection. Although peripheral thymocytes expressed the TCR complex at normal levels, anti-CD3ε stimulation triggered only a reduced proliferative response suggesting that the down-regulation occurred downstream of the TCR complex [21]. Injection of irradiated mice with bone marrow cells transduced with RhoH confirmed the crucial role of RhoH in thymocyte maturation and TCR signalling since reconstitution of RhoH compensated for the defects of RhoH-deficient mice.

**Figure 4:**
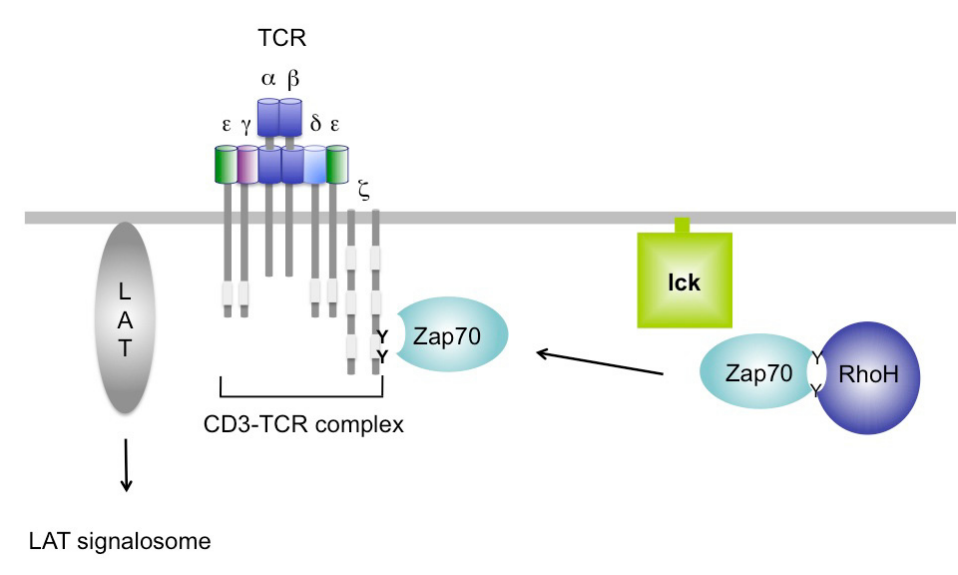
**RhoH is indispensable for correct TCR activation**. Illustration of the proposed mechanism that links RhoH to the active TCR complex. The ITAM-like motif of RhoH (see figure 1) allows binding of the Zap70 molecule and shuttling to the TCR CD3ζ chain. Zap70 binding to the CD3ζ chain eventually results in the activation of the linker for activation of T-cells (LAT) molecule and the so-called LAT signalosome. In the absence of RhoH, translocation of Zap70 to the immunological synapse and CD3ζ phosphorylation are impaired leading to reduced TCR-induced signalling and impaired thymocyte selection.

In order to identify the potential interaction partner of RhoH downstream of the TCR, a pulldown assay was performed using GST-coupled RhoH and lysates from Jurkat cells. A specifically interacting protein was analysed by mass spectrometry and found to be Zap70. Zap70 is a tyrosine kinase of the Syk family that is expressed in T-cells and NK-cells which interacts with the CD3ζ chain of the TCR complex [65]. The physical interaction of RhoH and Zap70 was confirmed in coimmunoprecipitation studies overexpressing the two proteins in HEK293 cells. Interestingly, this interaction was enhanced by additional expression of a Src family tyrosine kinase, Lck, which is known to be an activator of Zap70 (see Figure 4) [65]. Indeed, the authors found that stimulation of Jurkat cells with anti-CD3ε increased tyrosine phosphorylation of RhoH. However, while stimulation was a prerequisite for the interaction of tyrosine phosphorylated Zap70 with the TCR, the Zap70-RhoH complex was also detectable in unstimulated, resting cells. The consensus motif that is necessary for binding of Zap70 is a so-called immunoreceptor tyrosine based activation (ITAM) motif, characterised by two tyrosines, spaced by 9–11 amino acids: YXXL/I(X)_6–8_YXXL/I. A closer look at the RhoH sequence revealed that RhoH contains a sequence that the authors dubbed an ITAM-like motif, where the (iso)leucines at position +3 are replaced by an alanine. Interestingly, this motif is absent in any of the other Rho GTPases. Further mutational analysis confirmed the theory that the two tyrosines Y73 and Y83 in RhoH are essential for recruitment of Zap70. Deletion of the two SH2 domains in Zap70 known to bind to the TCR CD3ζ chain also abolished interaction with RhoH.

Like the RhoH-deficient mice, Zap70 null mice have a developmental block of the maturation at the double negative stage and peripheral thymocytes that are unresponsive to TCR mediated stimuli [66]. This suggests that RhoH plays a crucial role in TCR mediated Zap70 activity. Subcellular fractionation and immunofluorescence staining showed that RhoH acts as a shuttle for Zap70 and interacts with the pool of Zap70 molecules localised in the cytoplasm in the absence of antigen presenting cells. In the presence of an activating signal, the RhoH-Zap70 complex moves to the plasma membrane, where Zap70 then exchanges binding partners and interacts with the ITAM motif of CD3ζ instead. Zap70 mediated phosphorylation of the scaffold protein LAT and associated molecules such as SLP76 and PLCγ1, which together form the LAT signalosome, is crucial to initiate downstream events such as calcium influx or Erk activation (see Figure 4). Indeed, phosphorylation of LAT, VAV1 and of PLCγ ανδ Erk, were reduced in RhoH-deficient DP cells [21,28]. Despite the reduced activation of Vav1, the activity of Rac1 and Rac2 were normal after TCR activation. However, the authors noted that the basal level of Rac1 activity seemed to be elevated in RhoH-deficient T-cells, suggesting once more that RhoH is an antagonist for Rac1 activity by inhibiting basal Rac1 activity [28].

## Conclusion

The haematopoietic GTPase RhoH is an atypical family member of the Rho GTPases as it is constitutively active and not regulated through the classical cycling between GTP- and GDP-bound state. The protein was originally cloned as a fusion transcript with LAZ3/BCL6 in a non-Hodgkin lymphoma cell line [13] and has since been found to be mutated or translocated in a number of human cancers (see Figure 3). Since Ras is a well-known oncogene [67], it was anticipated that the novel Rho protein also had potential tumourigenic properties. Most data gathered so far however indicate that in human cancers the absence of a functional RhoH protein or reduced RhoH expression levels rather than its overexpression may play a role in pathogenesis [32,56,57]. Despite the search for activating mutations of the gene in haematopoietic malignancies, only mutations within the non-coding exons have been found. This indicates that RhoH has no transforming potential itself, but rather is an important regulatory molecule that must be expressed in order to protect the cell from malignant transformation. This is supported by a number of studies investigating the role of RhoH in signal transduction in haematopoietic cells, where RhoH often is associated with negative regulation of proliferation and migration and increased apoptosis. It was shown that RhoH is an important antagonist of Rac1, another Rho GTPase with a known function in cell transformation due to aberrant activation [68]. In a first attempt at understanding the signalling of RhoH in cancer cells, or rather its absence of signalling, it was shown that expression of RhoH is necessary to prevent Rac1 mediated protection from drug-induced apoptosis [56]. However, the function of RhoH is not restricted to antagonising Rac mediated signalling. RhoH-deficient mice showed an impaired development of thymocytes and reduced TCR signalling due to inefficient recruitment of Zap70 [21,28]. RhoH contains a novel ITAM-like motif that serves as a binding site for Zap70, which is then shuttled to the T-cell receptor complex where it serves as a crucial molecule to trigger activation of the LAT signalosome. Despite this important role of RhoH in T-cell development, it has not been addressed so far whether RhoH is also involved in T-cell specific malignancies, for example in T-cell lymphomas. Conversely, it is well established that RHOH is implicated in B-cell neoplasms, however, it is currently not known whether RhoH has a role in modulation of B-cell signalling.

Even though to date no other obvious defects of the RhoH-deficient mice have been reported, it is to be expected that RhoH will be an important regulatory molecule in a number of other signalling cascades. In an early study it was shown that stimulation of neutrophils with GM-CSF leads to an upregulation of RhoH mRNA, suggesting that RhoH may play a role in diseases characterised by neutrophilic inflammation such as chronic obstructive pulmonary disease (COPD) [69]. It will therefore be of immense interest to identify other regulators of RhoH expression and novel interacting proteins [70].

## List of abbreviations

GTP: guanosine 5'-triphosphate; GDP: guanosine 5'-diphosphate; GM-CSF: granulocyte-macrophage colony-stimulating factor; CNS: central nervous system.

## Competing interests

The authors declare that they have no competing interests.

## Authors' contributions

KFK wrote the manuscript and FF performed experiments and revised the manuscript. Both authors read and approved the final manuscript.
